# Cluster of Oseltamivir-Resistant and Hemagglutinin Antigenically Drifted Influenza A(H1N1)pdm09 Viruses, Texas, USA, January 2020

**DOI:** 10.3201/eid2707.204593

**Published:** 2021-07

**Authors:** Teena Mohan, Ha T. Nguyen, Krista Kniss, Vasiliy P. Mishin, Angiezel A. Merced-Morales, Jennifer Laplante, Kirsten St. George, Patricia Blevins, Anton Chesnokov, Juan A. De La Cruz, Rebecca Kondor, David E. Wentworth, Larisa V. Gubareva

**Affiliations:** Centers for Disease Control and Prevention, Atlanta, Georgia, USA (T. Mohan, H.T. Nguyen, K. Kniss, V.P. Mishin, A.A. Merced-Morales, A. Chesnokov, J.A. De La Cruz, R. Kondor, D.E. Wentworth, L.V. Gubareva);; General Dynamics Information Technology, Atlanta (T. Mohan, H.T. Nguyen);; New York State Department of Health, Albany, New York, USA (J. Laplante, K. St. George);; San Antonio Metropolitan Health District, San Antonio, Texas, USA (P. Blevins)

**Keywords:** drug resistance, neuraminidase inhibitor, antigenic drift, transmission, influenza, influenza A(H1N1), viruses, Texas, United States, antimicrobial resistance

## Abstract

Four cases of oseltamivir-resistant influenza A(H1N1)pdm09 virus infection were detected among inhabitants of a border detention center in Texas, USA. Hemagglutinin of these viruses belongs to 6B.1A5A-156K subclade, which may enable viral escape from preexisting immunity. Our finding highlights the necessity to monitor both drug resistance and antigenic drift of circulating viruses.

Resistance to antiviral drugs for influenza is an ongoing public health concern. The neuraminidase (NA) inhibitor oseltamivir is the most prescribed antiviral drug for controlling influenza. However, during 2007–2009, oseltamivir-resistant influenza A(H1N1) viruses rapidly spread worldwide (*1*). Molecular mechanisms implicated in this event were acquisition of NA-permissive mutations that alleviated deleterious fitness effects of the resistance-conferring mutation NA-H275Y (N1 numbering) (*2*); changes that improved balance of hemagglutinin (HA) and NA activities (*3*); and a “hitchhiking” mechanism, in which HA antigenic drift promoted the spread of oseltamivir-resistant viruses (*4*). Oseltamivir-resistant H1N1 viruses were later displaced by the 2009 pandemic virus, influenza A(H1N1)pdm09 (pH1N1), which was antigenically distinct and oseltamivir sensitive (*5*). The emergence and transmission of oseltamivir-resistant pH1N1 carrying a NA-H275Y mutation was first reported early in the 2009 pandemic (*6*). In the following years, transmission of oseltamivir-resistant viruses within healthcare settings and communities, or between close contacts, was occasionally observed (*1*); clusters were reported in Australia in 2011 (*7*) and Japan in 2013 (*8*). Despite these incidents, widespread circulation of oseltamivir-resistant viruses has yet to occur.

## The Study

The Centers for Disease Control and Prevention (CDC) receives influenza-positive specimens collected globally for virological surveillance. Viral genomes are analyzed using next-generation sequencing (NGS) to identify strains of epidemiologic, virologic, and clinical importance (*9*). To supplement US national antiviral surveillance, pyrosequencing is used by public health laboratories to screen additional viruses either in-house or by the National Influenza Reference Center (*10*).

During the 2019–20 influenza season, the pH1N1 subtype predominated in the United States. Later in the season, fewer influenza samples were identified, likely because of COVID-19 pandemic mitigation strategies. Of 951 pH1N1 isolates collected nationwide during October 2019–September 2020, 4 (0.4%) had the NA-H275Y marker. Supplemental surveillance, conducted on 282 viruses from 18 states collected November 2019–March 2020, detected another 6 (2.1%) NA-H275Y viruses, bringing the total detected nationwide to 10 (10/1,233; 0.8%). Of these, 4 (7.7%) were detected among 52 viruses from Texas. 

An investigation into a potential epidemiologic link revealed that these 4 virus isolates were collected from the same location, a border detention center in Webb County, Texas, on the same day (January 24, 2020). In January 2020, an influenza outbreak took place there; 8 cases were reported during January 19–28, 2020. All patients showed similar symptoms, such as fever, cough, sore throat, and body aches. Oseltamivir was prescribed on the same day, following specimen collection. Only 4 nasopharyngeal specimens from this outbreak were available for analysis; these samples were collected from men 25–59 years of age. San Antonio Metropolitan Health District Laboratory (San Antonio, TX, USA) conducted the initial diagnostic testing by real-time reverse transcription PCR and determined the cycle threshold (C_t_) values as 16.7–25.9, indicating relatively high viral loads. NGS analysis showed that the viruses had the oseltamivir resistance-conferring mutation, NA-H275Y. To expand testing, the San Antonio Laboratory submitted to CDC all remaining pH1N1 positive respiratory specimens (n = 36), collected from Webb County residents during November 2019–March 2020. These specimens were collected from 19 male and 17 female patients with a median age of 6 years (range 0–65 years); C_t_ values were 21.1–37.5. Pyrosequencing analysis concluded that there were no additional specimens with the NA-H275Y mutation.

A unique genomic signature can help in tracing the origin and spread of viruses in an outbreak. NGS analysis (*11*) revealed that the codon-complete genomes of the 4 cluster viruses were identical at a nucleotide level. Although the chain of transmission is unknown, considering the close-contact setting, this finding might suggest that an oseltamivir-resistant virus was transmitted from a single source. The cluster viruses shared 2 rare substitutions, PB1-Q687H and PB2-R251G, the combination of which was not found in other sequences from the National Center for Biotechnology Information and GISAID (https://www.gisaid.org; accessed October 22, 2020). Therefore, this virus has a unique genomic signature that has not been detected in viruses collected in Texas or elsewhere.

We isolated the 4 cluster viruses and propagated them in MDCK cells, followed by sequence confirmation. We tested the virus isolates for susceptibility to NA inhibitors using the NA inhibition assay (*10*) and they showed highly reduced inhibition by oseltamivir (≈1,300-fold) and peramivir (≈350-fold), and normal inhibition by zanamivir and laninamivir (Table 1). Markers associated with resistance to the polymerase inhibitor, baloxavir, were not detected. To confirm baloxavir susceptibility, we tested viruses by a high-content imaging-based neutralization test (HINT) (*12*). The concentrations of drug needed to inhibit infection by 50% fell in a low nanomolar range (mean 1.86 nM, SD 0.26), consistent with a susceptible phenotype.

**Table 1:** Susceptibility to NA inhibitors of influenza A(H1N1)pdm09 virus isolates from border detention center inhabitants, Texas, USA, January 2020*

Virus	NA resistance marker	Mean IC_50_ ± SD, nM (fold difference)			
		Zanamivir	Oseltamivir	Peramivir	Laninamivir
Test					
A/Texas/26/2020	H275Y	0.29 + 0.01 (2)	200.99 + 21.56 (1,182)	23.34 + 6.18 (333)	0.53 + 0.04 (2)
A/Texas/136/2020	H275Y	0.32 + 0.00 (2)	247.44 + 20.52 (1,456)	25.09 + 1.08 (358)	0.59 + 0.04 (3)
A/Texas/137/2020	H275Y	0.33 + 0.04 (2)	229.50 + 35.77 (1,350)	23.32 + 0.29 (333)	0.56 + 0.03 (3)
A/Texas/138/2020	H275Y	0.31 + 0.02 (2)	228.41 + 27.05 (1,344)	24.15 + 1.10 (345)	0.51 + 0.02 (2)
Reference					
A/Illinois/45/2019	Wildtype	0.19	0.17	0.07	0.22
A/Alabama/03/2020	H275Y	0.27 (1)	139.71 (822)	12.43 (178)	0.47 (2)

*The drug susceptibility of MDCK-grown viruses was determined using a NA inhibition assay. Reference viruses are from the Centers for Disease Control and Prevention Neuraminidase Inhibitor Susceptibility Reference Virus Panel version 3.0 (Atlanta, GA, USA). IC_50_ fold increase was determined by comparing to wild-type reference virus IC_50_. According to the World Health Organization Antiviral Working Group criteria, an increase below 10-fold constitutes normal inhibition, an increase of 10–100-fold is considered as reduced inhibition and an increase >100-fold is classified as highly reduced inhibition. IC_50_, concentration of drug needed to inhibit NA by 50%; NA, neuraminidase.

**Figure 1:**
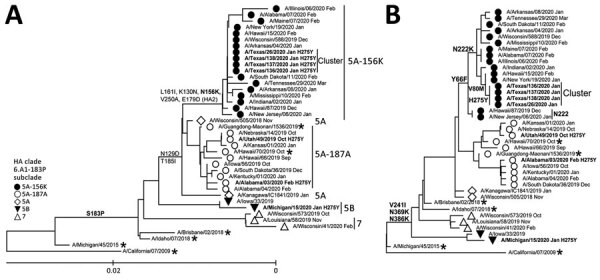
Evolutionary relationships of the HA (A) and NA (B) genes of influenza A(H1N1)pdm09 viruses circulating in the United States during the 2019–20 influenza season compared with reference viruses. We generated phylogenetic trees using MEGA software version 10.1.8 (http://www.megasoftware.net) and the bootstrap method (1,000 replications). We computed evolutionary distances by using the maximum composite likelihood model. Analysis included 40 representative A(H1N1)pdm09 HA and NA gene sequences. Boldface indicates oseltamivir-resistant viruses carrying NA-H275Y substitution; asterisks indicate vaccine viruses. A/California/07/2009 virus (the first A(H1N1)pdm09 vaccine) is used as a reference for ancestry (root) and numbering. Scale bar represents nucleotide substitutions per site. HA, hemagglutinin; NA, neuraminidase.

HA phylogenetic analysis placed the cluster viruses into the 6B.1A5A-156K subclade, which shares additional amino acid substitutions K130N, L161I, V250A in HA1 and E179D in HA2 (Figure 1, panel A). HA substitutions at residue 156 have been sporadically detected and shown to affect antigenicity, but no widespread circulation of such viruses was observed before summer 2019. In the United States, viruses with HA-5A-156K were first detected in fall 2019 and prevailed among pH1N1 by February 2020. Conversely, the circulation of another recently emerged subclade, 6B.1A5A-187A, 189E, had decreased by winter 2020. A/Hawaii/70/2019 virus (HI/70) from subclade 5A-187A, 189E, was selected as a pH1N1 vaccine component for the 2020–21 Northern Hemisphere influenza season; A/Wisconsin/588/2019 (WI/588), representing 5A-156K, was selected for the 2021 Southern Hemisphere vaccine.

We assessed the antigenicity of pH1N1 viruses representing distinct HA genetic groups circulating in the United States for antigenic relatedness by HINT and hemagglutination inhibition (HI) assays, using postinfection ferret antiserum (*13*,*14*). We used viruses A/Idaho/07/2019 (ID/07), HI/70, and WI/588, representing recent vaccines, and their homologous ferret antiserum as references. In the HINT assay, we found that the antiserum raised to ID/07 showed poor reactivity (65–78-fold reduction) to viruses with HA-N156K, including the cluster. The HI/70 antiserum reacted even more poorly (315–429-fold) against this group but maintained good reactivity to other HA groups. Antiserum raised to WI/588 (5A-156K) had very high titers against viruses of the same group, including the cluster, and reacted poorly (40–612-fold) to viruses of other groups (Table 2). Results obtained by the conventional HI assay corroborated the HA antigenic drift detected by HINT (Table 2). While analyzing antigenicity of pH1N1, it is prudent to consider that ferret antiserum may preferentially detect changes at HA antigenic site Sa, where N156K resides, compared with site Sb, where D187A is located (*15*). Nevertheless, the findings of this study and other reports indicate that viruses carrying HA-N156K may escape humoral immunity elicited by previous infections and vaccinations.

**Table 2:** Antigenicity of influenza A(H1N1)pdm09 viruses representing distinct HA genetic groups, United States, 2019–2020*

Virus	HA subclade 6B.1A	Ferret antiserum, titers (fold)					
		HINT assay			HI assay		
		ID/07	HI/70	WI/588	ID/07	HI/70	WI/588
Reference							
A/Idaho/07/2018	3	*28,421 * ***(1)***	23,464 **(3)**	543 **(250)**	*2,560 * ***(1)***	2,560 **(1)**	160 **(16)**
A/Hawaii/70/2019	5A-187A, 189E	15,132 **(2)**	*69,032 * ***(1)***	238 **(570)**	1,280 **(2)**	*2,560 * ***(1)***	160 **(16)**
A/Wisconsin/588/2019	5A-156K	592 **(48)**	552 **(125)**	*135,765 * ***(1)***	80 **(32)**	80 **(32)**	*2,560 * ***(1)***
Test							
n = 2	5B	60,007 **(2)**	83,978 **(1)**	3,402 **(40)**	2,560 **(1)**	5,120 **(1)**	Not tested
n = 7	7	37,410 **(1)**	35,135 **(2)**	784 **(173)**	2,560 **(1)**	2,560 **(1)**	80 **(32)**
n = 22	5A-187A, 189E	24,863 **(1)**	88,411 **(1)**	222 **(612)**	1,280 **(2)**	2,560 **(1)**	80 **(32)**
n = 21	5A-156K	399 **(71)**	190 **(363)**	207,958 **(1)**	320 **(8)**	320 **(8)**	5,120 **(1)**

*The antigenicity of MDCK-grown influenza A(H1N1)pdm09 viruses, representing different HA genetic groups, was tested by the HINT and HI assays, using postinfection ferret antiserum, generated from the influenza A(H1N1)pdm09 vaccine candidate viruses for the Northern Hemisphere during 2019–2020 (A/Idaho/07/2019; ID/07) and 2020–21 (A/Hawaii/70/2019; HI/70), and the Southern Hemisphere for the 2021 (A/Wisconsin/588/2019; WI/588) seasons. In the HINT assay, the neutralization titers were determined by calculating the antiserum dilution factor needed to reduce the infected cell population by 50% (average titers are shown) and the HI titers were expressed as the reciprocal of the highest serum dilution that inhibit the hemagglutination of 4 HA units of virus (median titers are shown). Italics indicate homologous titers; boldface indicates fold changes determined by comparing HINT or HI titers to the respective homologous titers. HA, hemagglutinin; HI, hemagglutination inhibition; HINT, high-content imaging-based neutralization test.

**Figure 2:**
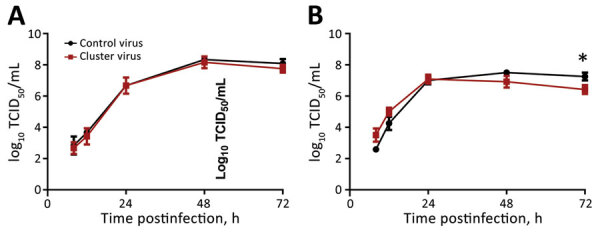
In vitro replicative fitness of influenza A(H1N1)pdm09 cluster and control viruses. The growth kinetics of the 2 viruses, the cluster virus A/Texas/137/2020, and A/New York/19/2020, were assessed using MDCK (A) and hCK (B) cell lines. These viruses have identical HA and NA amino acid sequences, except the H275Y substitution in NA. Cell monolayers were infected at a multiplicity of infection of 0.002 and the supernatants were harvested at 8, 12, 24, 48, and 72 hours postinoculation. Infectious virus titers were determined and expressed as log_10_ TCID_50_/mL. The lower limit of virus detection is 1.75 log_10_ TCID_50_/mL. Data are shown as mean +SD; we used the unpaired *t*-test with Welch’s correction for statistical comparisons (asterisk indicates p˂0.05). The hCK cell line was kindly provided by Dr. Y. Kawaoka (University of Wisconsin, Madison, WI, USA) per material transfer agreement. HA, hemagglutinin; hCK, humanized MDCK cells; NA, neuraminidase; TCID_50_, median tissue culture infectious dose.

We assessed the in vitro replicative fitness of the cluster virus A/Texas/137/2020, and A/New York/19/2020, which has identical HA and NA amino acid sequences except for NA-H275Y. These 2 viruses had very similar growth kinetics in MDCK and humanized MDCK (hCK) cells. In MDCK cells, the growth curves were alike at all time points (Figure 2, panel A). In hCK cells, the NA-H275Y-containing virus had better growth at 8 hours, but its titers tapered off slightly at later times (Figure 2, panel B).

Phylogenetic analysis of NA (Figure 1, panel B) showed that the cluster viruses had NA similar to the majority of viruses in the 5A-156K group, including characteristic substitutions NA-Y66F and NA-N222K. However, their NA contained a rare substitution, NA-V80M. Studies to evaluate the effects of these changes on HA–NA functional balance are ongoing.

## Conclusions

Although no evidence of oseltamivir-resistant virus transmission outside the detention center was found, the properties of the cluster viruses are concerning. They belong to an HA antigenically drifted group, and escape from preexisting immunity may contribute to the spread of oseltamivir-resistant viruses in coming seasons.
